# Methods and Applications of Lanthanide/Transition Metal Ion-Doped Luminescent Materials

**DOI:** 10.3390/molecules30173470

**Published:** 2025-08-23

**Authors:** Xiaoyi Chen, Jiaqi Liu, Shujing Zhou, Zan Li, Min Yuan, Jinghui Shen, Yifan Zhang, Rongrong Ye

**Affiliations:** 1 School of Pharmacy, Jiamusi University, Jiamusi 154007, China; cxy84828@126.com (X.C.); 15946326871@163.com (J.L.); zhshj2003@163.com (S.Z.); lizan4432@163.com (Z.L.); m15645326791@163.com (M.Y.); 15045118693@139.com (J.S.); 13069758081@139.com (Y.Z.); 2 Architectural Engineering Institute, Jiamusi University, Jiamusi 154007, China

**Keywords:** Lanthanide/transition metal, luminescent materials, synthesis techniques, applications

## Abstract

Lanthanide/transition metal-doped luminescent materials are advanced materials with broad application potential. This type of material achieves control and optimization of luminescence performance by introducing lanthanide/transition metal ions into the host material and utilizing its unique electronic structure and optical properties. Luminescent materials are suitable for optical communication devices, biological imaging, and photodetectors. The combination of lanthanide/transition metals with various matrix materials provides a new platform for creating new chemical and physical properties in materials science and device applications. In this paper, we summarize the latest progress in the research of lanthanide/transition metal-doped luminescent materials and explain their roles in biological imaging, sensing, and optoelectronic applications. It starts with various synthesis techniques and explores how to cleverly incorporate rare earth/transition metals into various matrices, thereby endowing them with unique properties. Then, the advantages and disadvantages of each synthesis technique are discussed. Subsequently, the focus will be on functional strategies and their applications. Finally, strategies for lanthanide/transition metal ion-doped luminescent materials to address challenges are proposed, and insights from each section are summarized.

## 1. Introduction

Luminescent materials have always been the foundation and hot topic of various research and application studies due to their wide range of applications. In addition to the well-known applications in the field of lighting, significant breakthroughs have been made in the application of optoelectronic devices, display devices, and other fields. Lanthanide and transition metal ion-doped inorganic luminescent materials have attracted widespread attention due to their excellent optical properties. Compared to traditional organic dyes and quantum dots, rare earth/transition metal-doped inorganic luminescent materials have tunable luminescence, the ability to achieve near-infrared excitation and emission, low toxicity, excellent photostability, no photobleaching, and a long luminescence lifetime. In addition, near-infrared emission has high tissue penetration depth and low autofluorescence and tissue scattering. Therefore, in recent years, breakthroughs have been made in the application research of lanthanide/transition metal-doped inorganic luminescent materials, such as anti-counterfeiting, temperature sensing, optical information storage, lighting display, and biomedicine [1,2,3,4,5]. Fluorescent materials have many applications in the field of architecture. People can use lanthanide or transition metal oxide fluorescent materials to make evacuation signs. Under normal circumstances, these signs are no different from ordinary signs, but in emergency situations, such as power outages or fires, fluorescent materials can emit light, providing clear evacuation guidance for people and improving the efficiency and safety of personnel evacuation. Lanthanide and transition metal oxide fluorescent materials can be added to interior decoration materials such as coatings, wallpapers, and panels. During the day, these materials may present ordinary colors or textures, but at night or in low-light environments, they can emit unique fluorescence, creating a magical and romantic atmosphere for indoor spaces. In scenarios requiring enhanced directional guidance, such as age-friendly environments, these materials can significantly improve the visibility and recognizability of spatial orientation, showcasing functional advantages that traditional materials may not provide (such as SrAl_2_O_4_:Eu^2+^ and Dy^3+^). Traditional luminescent materials refer to a type of luminescent material that was developed, widely used, and technologically mature in earlier times. They mainly generate light radiation through excitation, such as light, electricity, and cathode rays, and have long-term applications in fields such as lighting, display, and detection [6].

Near-infrared luminescent materials doped with rare earth transition metal ions exhibit many remarkable characteristics. First, they have bright near-infrared luminescence, which makes them widely applicable in fields such as displays, lighting devices, and biological imaging technology. Second, these materials have good stability and a long lifespan and can maintain stable luminescence under continuous excitation. In addition, by selecting appropriate rare earth ions and transition metal ions, fine control of the luminescent color of the material can be achieved [7,8].

The luminescence of lanthanide and transition metal oxides depends on external energy excitation, which causes ion electrons to transition from the ground state to the excited state, and then releases photons through the transition. The common ways of stimulation are as follows:

(1). Photoinduced excitation (most common)

Principle: Materials absorb light of specific wavelengths (such as ultraviolet and blue light), and energy is transferred to luminescent ions, causing their electrons to transition.

Example: YAG:Ce^3+^ phosphor in LED absorbs the blue light emitted by the chip, excites Ce^3+^, and releases yellow light, which mixes with the remaining blue light to form white light. The Eu^3+^-doped oxide in anti-counterfeiting ink emits red fluorescence after absorbing ultraviolet light.

(2). Electro-excitation

Principle: Through the action of an electric field, electrons or holes migrate in the material and collide with luminescent ions, transferring energy to excite them.

Example:

Electroluminescent devices (such as lanthanide complexes in OLEDs) excite luminescent ions through charge carriers injected into the electrode.

Transition metal oxides in plasma display panels (PDPs) emit light due to electron bombardment generated by plasma discharge.

(3). Cathodic ray excitation

Principle: High-energy electron beams (cathode rays) directly bombard materials, and the kinetic energy of electrons is transferred to luminescent ions to excite them.

Example: Fluorescent powders (such as ZnO:Zn and transition metal oxides) in traditional CRT displays are excited by electron beams to emit light and form images.

Electron microscope fluorescent screens use lanthanide oxides to convert electronic signals into visible light.

(4). Thermal excitation (thermoluminescence)

Principle: When a material is heated, the lattice vibration energy is transferred to luminescent ions, or electrons captured by traps are released, triggering transition luminescence.

Example:

Li_2_B_4_O_7_:Mn^2+^ (transition metal-doped) in a thermoluminescence dosimeter is used to detect radiation dose through thermal luminescence intensity.

Some anti-counterfeiting labels emit specific colored fluorescence when exposed to heat.

(5). Chemical excitation (chemiluminescence)

Principle: The energy released by chemical reactions (such as oxidation–reduction reactions) is transferred to luminescent ions, causing them to excite and emit light.

Example:

In some chemical sensors, transition metal oxides (such as Co₃O₄) react with specific gases to release energy, triggering fluorescence changes for detection.

The luminescent materials doped with rare earth and transition metal ions usually adopt the high-temperature solid-phase method, sol-gel method, chemical vapor deposition method, etc. These methods require precise control of reaction conditions to ensure the high quality and reproducibility of the materials. Among them, the high-temperature solid-phase method is the most commonly used method because it can provide high-purity samples and good crystallinity. However, for some temperature-sensitive materials, such as those containing organic ligands, the sol-gel method may be more appropriate [9,10,11,12].

The luminescent core of lanthanide and transition metal oxides is the release of energy through electronic transitions, which is closely related to their ionic properties. Lanthanide oxides: The 4f electron layer of lanthanide elements (such as Eu^3+^, Tb^3+^, and Ce^3+^) is shielded by outer electrons, and the energy level structure is stable. The luminescence mainly comes from electron transitions within the 4f orbitals (such as the ^5^D_0_→^7^F_2_ transition of Eu^3+^ emitting red light). Transition metal oxides: The 3d electron layer of transition metal ions (such as Mn^2+^ and Cr^3+^) is unshielded, and their energy levels are easily affected by the matrix crystal field. The luminescence originates from 3d orbital electron transitions [13].

Rare earth elements include lanthanide elements (La-Lu) as well as scandium (Sc) and yttrium (Y), and their ions typically exist in a^3+^ valence state (such as Eu^3+^, Tb^3+^, Er^3+^, etc.). The core characteristic of rare earth ions is an unfilled 4f electron shell, which is shielded by the outer 5s^2^ and 5p^6^ electrons. This shielding effect makes 4f electrons less affected by external crystal fields and their energy level structure relatively stable.

(1). 4f electronic configuration: The 4f electron number of rare earth ions varies from 0 (La^3+^) to 14 (Lu^3+^), resulting in a complex and diverse energy level structure. The angular quantum number l of the 4f orbital is 3, and the magnetic quantum number m can range from −3 to +3, with a total of seven orbitals and a maximum capacity of 14 electrons.
(2). Energy level splitting: The 4f energy level of a free rare earth ion splits into multiple energy levels (such as ^2^S + ^1^L-J) due to spin–orbit coupling. In crystals, the crystal field further causes energy level splitting, but due to the shielding of 4f electrons, the degree of splitting is relatively small (usually several hundred wavenumbers).

The luminescence of rare earth ions mainly originates from the transitions of 4f electrons between different energy levels, including f-f transitions and f-d transitions.

(1). f-f transition: Mechanism: 4f electrons transition within the same shell (Δl = 0). Electric dipole transitions are originally forbidden, but due to the symmetry breaking of the crystal field or mixing with configurations of opposite parity, the transition is allowed. Characteristics: The spectrum is sharp and linear, with a high color purity. Low transition probability and long excited state lifetime (in milliseconds) are called metastable states. The emission wavelength is determined by the rare earth ions themselves and is less affected by the external environment.
(2). f-d transition: Mechanism: 4f electrons transition to 5d orbitals (Δl = 1), allowing for electric dipole transitions. It is commonly found in low-valence rare earth ions (such as Ce^2+^, Eu^2+^, and Yb^2+^). Characteristics: The spectrum exhibits broadband, high intensity, and a short fluorescence lifetime (nanosecond level). The emission wavelength is significantly affected by the crystal field.
(3). Charge transfer band (CTS): Electrons migrate from the molecular orbitals of ligands (such as O^2−^) to the 4f orbitals of rare earth ions, forming a broad band absorption. It is commonly used to enhance the light absorption efficiency of rare earth ions.

Due to their unique luminescent properties and excellent stability, luminescent materials doped with rare earth and transition metal ions have broad application prospects in many fields. First, they can be used to manufacture high-performance and long-lasting displays and lighting devices (such as YAG:Ce^3+^ and CaAlSiN_3_:Eu^2+^). Second, due to their near-infrared luminescence properties, they can also be used in biological imaging techniques such as fluorescent probes and biological tissue imaging (such as UCNPs and DCNPs). In addition, these materials can also be used for energy conversion and storage, such as electrode materials for solar cells and batteries (such as DSSCs) [14].

Examples of luminescent materials for commercial applications:

(1). LED lighting and display

Yttrium Europium Oxide (Y_2_O_3_:Eu^3+^): As a red fluorescent powder, it is used in conjunction with blue LED chips to achieve white LED for indoor lighting and display backlighting.

(2). Imaging and visualization

LaGdO_3_: Eu^3+^: Used for X-ray intensifying screens to enhance imaging clarity.

Titanium oxide-doped transition metals (such as TiO_2_:Cr^3+^) are used as fluorescent probes for biomedical imaging.

(3). Security and anti-counterfeiting measures

Strontium dysprosium oxide (SrAl_2_O_4_:Dy^3+^ and Eu^2+^): a long-lasting phosphorescent material used for emergency signs and luminous safety signs. It can emit light for more than 10 h in the dark without the need for a power source.

Dysprosium oxide-doped materials (such as ZnO:Dy^3+^) can be used for anti-counterfeiting inks, displaying hidden fluorescent patterns through specific wavelength excitation [15,16].

In this review, we explored the latest developments in the synthesis and application of lanthanide/transition metal ion- activated luminescent materials. Our exploration spans from high-temperature phase synthesis technology to hydrothermal synthesis, exploring the advantages and disadvantages of various technologies, as well as their applicability. We emphasize the importance of functionalization in utilizing its unique properties and strategies to expand the applications of these materials, particularly in the fields of biological imaging and biomedical applications, where it has advantages in deep tissue penetration and minimal biological interference. Through extensive and in-depth discussions on relevant research, the development of both basic research and technological applications of lanthanide/transition metal-doped luminescent materials can be promoted [17,18,19,20,21].

## 2. Technology and Methods of Lanthanide/Transition Metal Ion-Doped Luminescent Materials

### 2.1. Preparation of Luminescent Materials by the High-Temperature Solid-State Method

The high-temperature solid-phase synthesis method refers to the formation of a large number of composite oxides through contact, reaction, nucleation, and crystal growth reactions between solid interfaces at high temperatures (1000~1500 °C). The high-temperature solid-phase method is a commonly used method for preparing luminescent materials, which includes the basic steps of raw material selection, pretreatment, grinding and mixing, high-temperature calcination, and post-treatment [22,23,24].

Misevicius M prepared single-phase europium (Eu_2_O_3_)-doped and dysprosium co-doped SrAl_4_O_7_ samples using the solid-state reaction method and confirmed the phase purity of the synthesized sample by powder X-ray diffraction measurement. The sample doped with europium exhibited blue-green luminescence at 490 nm, while co-doping with dysprosium shifted the maximum emission to 475 nm, thus shifting the color towards the blue region. The influence of dopant concentration and molar ratio on the luminescence properties of strontium aluminate was studied [25].

Song Y synthesized a series of La_2_LiSbO_6_ phosphors doped with Mn^4+^ and Eu^3+^, as well as co-doped with Eu^3+^/Mn^4+^. Detailed characterization and analysis were conducted on the crystal phase and optical properties of these materials. Experimental analysis showed that La_2_LiSbO_6_:Eu^3+^/Mn^4+^ fluorescent powder had relatively high temperature sensitivity and potential application prospects in the field of high-temperature sensing [26].

Pavani K reported a series of Tb^3+^- and Eu^3+^-activated SrMg_2_La_2_W_2_O_12_ phosphors synthesized using the solid-state reaction method. By changing the concentration and excitation wavelength of phosphors doped with Tb^3+^ and Eu^3+^ ions, the emission color can be altered to obtain white light emission. The International Commission on Illumination’s (CIE) chromaticity coordinates and correlated color temperature (CCT) indicated that when SrMg_2_La_2_W_2_O_12_:Tb^3+^Eu^3+^ phosphor is excited by ultraviolet (UV) light, it can be modulated to emit white light at 450 to 750 nm [27].

**Figure 1:**
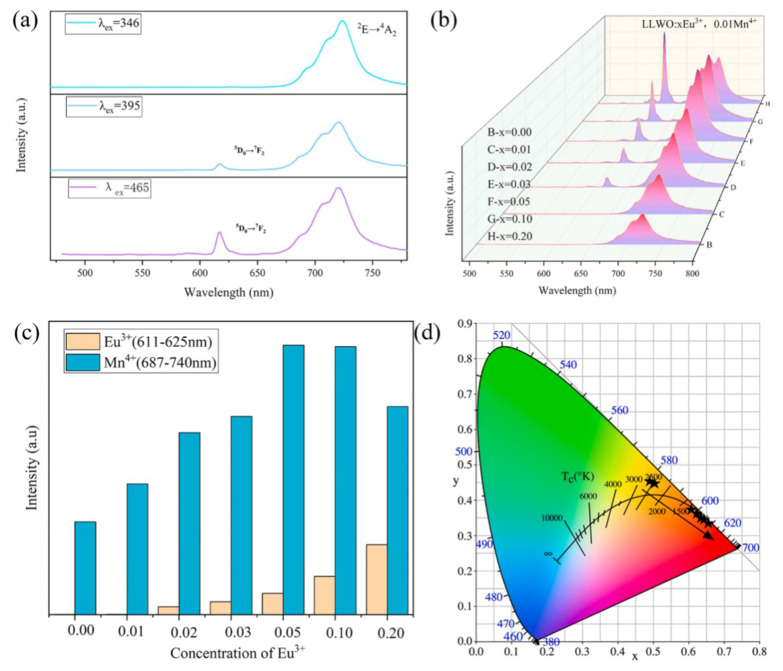
(**a**) PL spectra of LLWO:0.05Eu^3+^ and 0.01Mn^4+^ phosphor excited at 346 nm, 395 nm, and 465 nm. (**b**) PL spectra of LLWO:xEu^3+^ and 0.01Mn^4+^ samples excited at 465 nm. (**c**) Integrated emission intensities of Mn^4+^ and Eu^3+^ ions with different doped Eu^3+^. (**d**) CIE chromaticity map corresponding to the prepared phosphors [28]. Copyright 2022. Reproduced with permission from Elsevier.

Tang Q obtained a family of La_3_Li_3_W_2_O_12_ (LLWO) red luminescent phosphors co-doped with rare earth (Eu^3+^) and transition metal (Mn^4+^) through the design of a placeholder strategy. By changing the concentration of Eu^3+^ and adjusting the coordination environment of the activator, the adjustable emission of fluorescent powder from orange to red was ultimately achieved (Figure 1) [28].

Zhang prepared a novel visible red long afterglow luminescent material using a high-temperature solid-state method by adjusting the doping concentration of Bi^2+^ and changing the calcination temperature. When the doping concentration of Bi^2+^ was 0.50% and the calcination temperature was 1150 °C, the prepared Sr_3_Y_2_Ge_3_O_12_:Bi^2+^ exhibited the best luminescence performance. After being excited by 254 nm ultraviolet light, the material exhibited red afterglow luminescence intensity, duration, and stability that met practical application requirements. The trap characteristics and internal mechanisms of long afterglow were comprehensively studied through thermoluminescence, photoluminescence, and fluorescence spectroscopy [29].

Wang developed a high-temperature solid-state restricted growth strategy to prepare highly emissive CsPbBr_3_ nanocrystals using in-situ formed mesoporous Al_2_O_3_ as a template. The CsPbBr_3_-Al_2_O_3_ powder obtained has a quantum yield of up to 70%, a narrow emission linewidth of 25 nm, and excellent thermal stability. In addition, a batch of 500 g of CsPbBr_3_-Al_2_O_3_ powder was produced, demonstrating the potential for large-scale production using this method [30].

As a non-equilibrium amorphous substance between solid and liquid, glassy materials, among which hybrid metal halide glasses are particularly noteworthy, exhibit novel physical and chemical properties different from crystalline materials while retaining the crystalline coordination structure and special optical functions.

**Figure 2:**
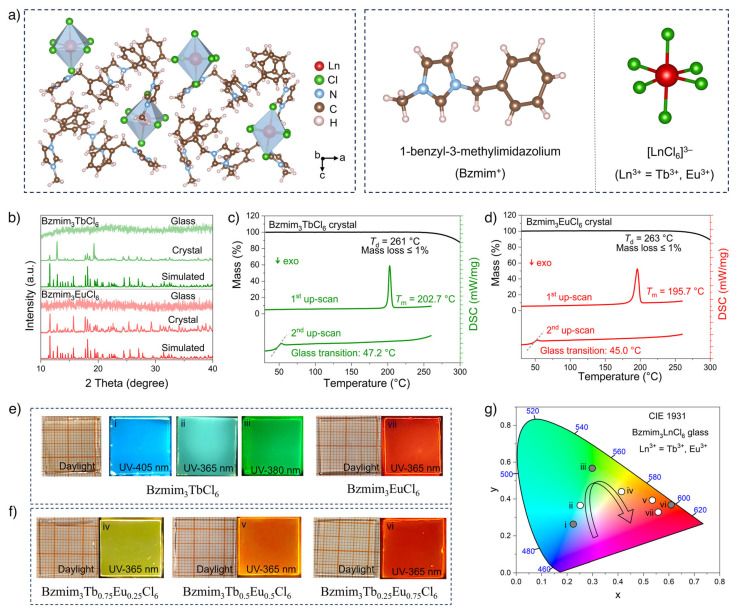
Structural and thermodynamic properties of Bzmim_3_LnCl_6_ (Ln^3+^ = Tb^3+^, Eu^3+^) crystals. (**a**) Crystal structure of Bzmim_3_LnCl_6_ and the corresponding localized structure. (**b**) Comparison of simulated and experimental PXRD patterns of Bzmim_3_LnCl_6_. (**c**,**d**) TG and DSC curves of Bzmim_3_LnCl_6_ crystals. (**e**–**g**) Luminescence picture of Bzmim_3_LnCl_6_ glass and the corresponding chromaticity coordinates [31]. Copyright 2025. Reproduced with permission from Wiley.

Researchers have prepared a novel amorphous organic–inorganic hybrid rare earth halide luminescent glass material, i.e., Bzmim_3_LnCl_6_ (Bzmim = 1-benzyl-3-methylimidazole; Ln^3+^ = Tb^3+^, Eu^3+^), and achieved tunable multi-color photoluminescence emission. By adjusting the doping ratio of Tb^3+^ and Eu^3+^ in Bzmim_3_LnCl_6_ glass, controllable radiative luminescence properties from green to red were successfully achieved under X-ray excitation (Figure 2) [31]. The author successfully prepared pure-phase Cs_2_Ag_1−x_Na_x_BiCl_6_ (x = 0, 0.25, 0.50, 0.75, 0.90, 0.95, 1) single-crystal materials using a solvothermal synthesis method (peak value = 700 nm, luminescence quantum efficiency = 51%, full width at half maximum = 270 nm, and Stokes shift = 340 nm) [32].

High-temperature solid-state synthesis is considered a more environmentally friendly process for preparing perovskite nanocrystals than colloidal synthesis. However, controlling the size and size distribution of nanocrystals at high temperatures is relatively difficult, which often leads to lower photoluminescence quantum yield (PLQY) and wider emissions. Zhang Q successfully prepared a highly stable CsPbBr_3_ nanocrystal with a PLQY of up to 93% and a narrow emission linewidth of 18 nm through solid-state synthesis using a hard template at a high temperature (600 °C). Research has found that the thermal stability of nanocrystals in mesoporous silica (MS) templates and the precise confinement of growth in uniform pores are the main reasons for achieving high PLQY and narrow emission, and the pre-hydrolysis sealing strategy helps to achieve excellent stability [33].

Yang X developed a new high-temperature mechanochemical (HTMC) method to synthesize single-phase Sr_2_CeO_4_:Eu^3+^ phosphors. Compared to the fluorescent powder prepared using the traditional high-temperature solid-phase method and the citric acid gel method, the single-phase Sr_2_CeO_4_:Eu^3+^ powder prepared using the HTMC method had an average particle size of about 5 µm, a narrow particle size distribution range, and uniform dispersion. It was prepared at 800 °C and reached the maximum luminous intensity at 900 °C. Under UV excitation at 298 nm, the sample exhibited good luminescence performance, with the strongest red light at 616 nm [34].

Compounds with a garnet structure exhibit excellent performance in fields such as fluorescent luminescent materials and are very promising compounds. A new blue light-excited yellow-emitting fluorescent powder, BaLu_2_ (Mg_0.6_Al_2.8_Si_1.6_) O_12_ Ce^3+^, was prepared by Wang. The external quantum efficiency (EQE) was 66.2%. At the same time as the redshift of Ce^3+^ emission, this substitution also widens the emission of Ce^3+^ and reduces its thermal stability [35].

The advantages of the high-temperature solid-state synthesis method for preparing luminescent materials are simple operation, low cost, and suitability for large-scale production. In addition, the prepared luminescent materials have stable performance, and the preparation method is relatively environmentally friendly, without solvent pollution problems. The shortcomings of this method are a high reaction temperature, high energy consumption, and the significant impact of raw material particle size, purity, and morphology on the reaction rate and luminescence performance. One direction for the future is the combination of physical high-temperature solid-phase and chemical methods, which not only save costs and improve efficiency but also produce fluorescent powders with a uniform particle size and have a lower tendency to agglomerate [36].

### 2.2. Preparation of Luminescent Materials by the Sol-Gel Method

The sol-gel method uses compounds containing highly chemically active components as precursors, uniformly mixes these raw materials in the liquid phase, and carries out hydrolysis and condensation chemical reactions to form a stable transparent sol system in the solution. The sol slowly polymerizes between aging colloidal particles to form a three-dimensional network structure of the gel. The gel network is filled with a solvent that has lost liquidity, forming a gel. The gel is dried, sintered, and solidified to prepare materials with molecular and even nanostructures [37].

For this reason, the commonly adopted strategy includes embedding the light-emitting object species into the normally non-light-emitting sol-gel host material, so as to form an optical system that retains both the optical physical characteristics of the object and the mechanical and morphological characteristics of the host [38,39].

Gökçe S used stoichiometric yttrium nitrate and aluminum nitrate as substrates to synthesize a phosphor material with the molecular formula of YAlO_3_ using the sol-gel method. The fluorescent powder obtained was doped/co-doped with Tb^3+^ and Nd^3+^ rare earth ions, as well as Mn_2+_ transition metal ions. Strong green emission was detected in the Mn^2+^-doped sample, corresponding to the 4T1 (G)→6A1 (S) transition. For YAlO_3_:Nd^3+^, characteristic emissions originating from 4G11/2→4I9/2 (∼423 nm), 4G9/2→4I49/2 (≈460 nm), and 4G7/2→4I9/20 (W 540 nm) transitions were observed. The optical bandgap of the undoped sample was calculated to be 2.79 eV, which decreases to the range of 2.46–2.56 eV based on the presence of Nd^3+^- and Tb^3+^-doped ions [40].

**Figure 3:**
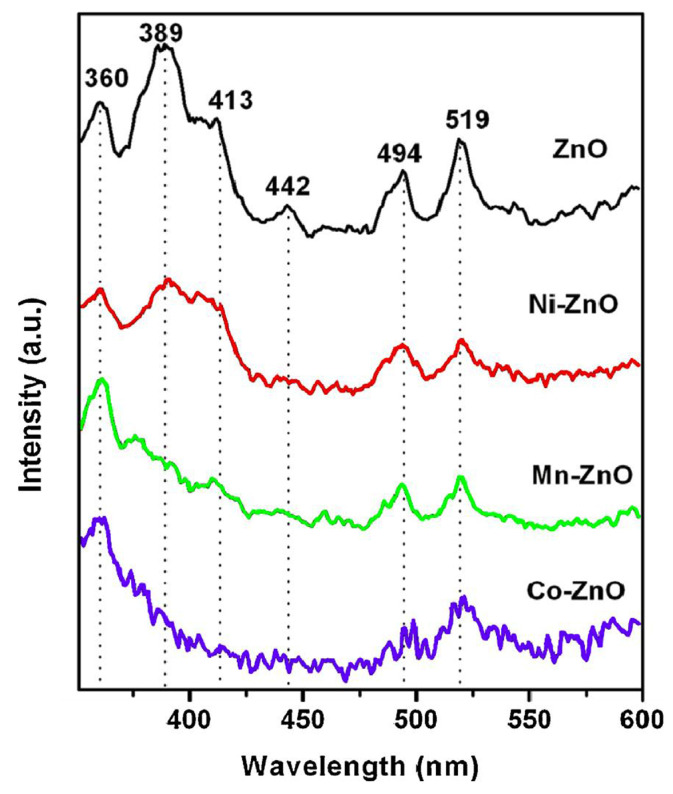
PL spectra of pure ZnO and TM (Ni, Mn, and Co)-doped ZnO thin films at room temperature [41]. Copyright 2014. Reproduced with permission from Elsevier.

Vijayapraath G prepared pure zinc oxide (ZnO) films doped with transition metals (TM = Ni, Mn, Co) using the sol-gel spin coating method, and the concentration of transition metals was 0.03 mol%. X-ray diffraction studies revealed the polycrystalline properties of the film, including the presence of a hexagonal wurtzite structure. UV transmission spectra indicated that all films were highly transparent in the visible light region. In the case of doped ZnO films, d-d transitions were observed in the purple region due to the presence of crystal defects and grain boundaries. Due to the orbital splitting of magnetic ions, the optical bandgap of the thin film decreased as the number of 3D electron orbitals occupied increased. The observation of ultraviolet and near-infrared electronic transitions indicated a strong relationship between transition metal doping and ZnO sites (Figure 3) [41].

El Ghoul J prepared Zn_2_SiO_4_ and Zn_2_SiO_4_:Mn particles embedded in a SiO_2_ matrix using the sol-gel method in two steps under an ethanol supercritical condition. After doping ZnO and ZnO: Mn nanoparticles into a single block of silica, they were prepared using a simple solid-state reaction at 1200 °C in a natural atmosphere. In the case of SiO_2_/Zn_2_SiO_4_ nanocomposites, powders with an average particle size of 80 nm exhibited a strong luminescent band centered around 760 nm in the visible light range. In addition, the PL spectrum of SiO_2_/Zn_2_SiO_4_: Mn nanocomposite material showed a main peak at 525 nm, which originated from the 4T1-6A1 transition of Mn^2+^ ions [42].

Talane T reported the upconversion and down-conversion luminescence behavior of anatase erbium-doped titanium dioxide synthesized by the sol-gel method. When excited at 320 nm, two down-conversion contributions of indirect bandgap and defect-related emission were observed at 378 nm and 435 nm, respectively, from pure and Er^3+^-doped TiO_2_ nanoparticles. On the other hand, under 980 nm laser excitation, strong green upconversion emission centered at 544 nm was observed in all TiO_2_ samples doped with Er^3+^, attributed to the 4S3/2→4I15/2 transition of Er^3+^. This analysis provided insights into the structural, optical, and luminescent properties of TiO_2_ nanoparticles doped with Er^3+^ for use in solar cells and biological imaging devices [43].

Szpikowska Roka studied the luminescent properties of Tb^3+^-doped silica sol-gel nanomaterials. The new experimental results of a silica sol-gel glass system containing a terbium-doped nanocrystalline β-cubic PbF_2_ phase were introduced. The study mainly focused on the effects of silica matrix composition, excitation wavelength, and heat treatment process on the luminescence properties of prepared silica samples. Based on excitation and emission measurements, as well as luminescence attenuation analysis, some spectral parameters of Tb^3+^ were successfully determined. The composition of the studied material changed while maintaining a constant concentration of terbium ions. The emission band intensity and luminescence decay curve of Tb^3+^ ions depended on the excitation wavelength. The obtained results indicated that Tb^3+^ ions were doped into the nanocrystalline phase during the ceramicization process [44].

Lauria A proposed a strategy for controlling the structure and optical properties of inorganic luminescent oxide (HfO_2_)-based nanoparticles. The nonaqueous sol-gel route was found to be suitable for the synthesis of hafnium nanoparticles and their doped rare earth (RE) ions, which would make them glow under ultraviolet and X-ray irradiation. In addition, the research team also revealed the ability of this technology to achieve low-temperature stability of a cubic phase by effectively doping trivalent rare earth ions into the lattice. For identical concentrations of Er^3+^ and Yb^3+^, the comparison of the cubic and monoclinic polymorphs of HfO_2_ showed the essential role of the crystal structure alone in the upconversion luminescence (UCL) [45].

**Figure 4:**
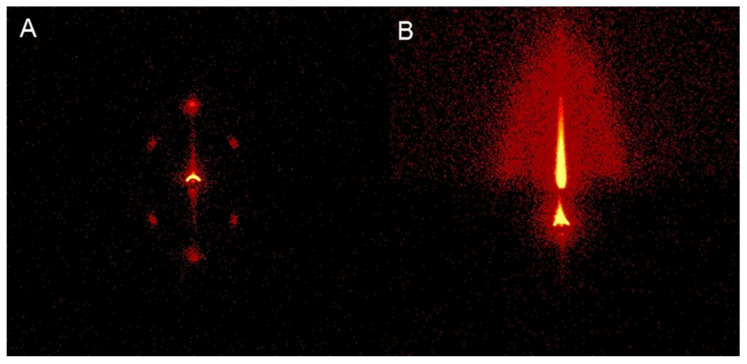
GISAXS pattern of (**A**) a pure TEOS-coated silicon wafer and (**B**) a wafer coated with the pure single-source precursor SSP2-Eu (Si/Eu = 3/1) [46]. Copyright 2012. Reproduced with permission from the American Chemical Society.

In the presence of Pluronic P123 as a structural directing agent, Feinle A prepared mesoporous silica coatings containing europium (III) ions using spin coating technology and the solvent evaporation-induced self-assembly (EISA) method with different single-source precursors (SSPs). By processing a mixture of tetraethyl orthosilicate (TEOS) and Eu3+coordinated SSPs, the chemical composition of porous coatings with different Si: Eu ratios was carefully customized. The photoluminescence spectrum clearly displayed characteristic emission peaks corresponding to the 5D0→7FJ (J = 0–5) transition, resulting in visible red luminescence to the eye, despite the very low thickness of the film (150–200 nm) (Figure 4) [46].

Rosa A synthesized Tm^3+^-doped white luminescent borosilicate glass ceramics using the sol-gel method. This characteristic photoluminescence emission was due to the 1D2→3F4 electron transition of Tm^3+^ ions and the π-π* and n-π* transitions of Cd double-bond C and Cd double-bond O caused by carbon residues inside the glass ceramic. The released white emission had a correlated color temperature of 5600–6650 K, a color rendering index of approximately 88, and a quantum yield of 12.3% [47].

Keskin successfully synthesized undoped Tb^3+^ and Dy^3+^-doped LiMgPO_4_ phosphors using the sol-gel method. In addition, the influence of Tb^3+^ and Dy^3+^ ions in LiMgPO_4_ on the luminescence intensity was also measured in detail. When the fluorescent powder was irradiated with ultraviolet light (254 nm), the main thermoluminescence emission peak was observed at 288 °C for Dy^3+^-doped phosphors, while peaks were observed at 198 °C and 283 °C for Tb^3+^-doped phosphors [48].

Gahlaut synthesized ultra-small copper-doped ZnO nanostructures with high luminescence by the ultrasonic sol-gel method. The observation of weak broadened peaks in X-ray diffractograms indicated the formation of a nanocrystal made of very small ZnO. Self-assembling nanostructures were obtained on glass substrates using a spin coating machine. The doping of copper not only enhanced the photoluminescence peak in the ultraviolet region but also produced strong yellow, green, and blue luminescence peaks. The strong green luminescence and weak ferromagnetism of ZnO nanowires made them suitable for magnetic, optoelectronic, and biosensor devices [49].

A series of red luminescent Ca_2_-xAl_2_SiO_7_:xEu^3+^(x = 1 mol%–10 mol%) phosphors was synthesized by the sol-gel method for Cai. The effects of annealing temperature and doping concentration on the crystal structure and luminescence properties of Ca_2_Al_2_SiO_7_:Eu^3+^ fluorescent powder were studied. After annealing at 1100 °C, the highest photoluminescence PL intensity was observed at an Eu^3+^ concentration x = 7 mol% [50].

The advantages of the sol-gel method include the following:

Homogeneous mixing at the molecular level: The sol-gel method can obtain molecular-level uniformity in a very short time through the solution reaction step, and the reactants are likely to be evenly mixed at the molecular level when forming a gel. Easy to dope trace elements: Through the solution reaction step, some trace elements can be uniformly and quantitatively doped, achieving uniform doping at the molecular level [51,52,53].

Lower synthesis temperature: Compared to the solid-phase reaction, the chemical reaction in the sol-gel method is easier to carry out, and the required temperature is lower, because the diffusion of components occurs in the nanometer range. Preparation of new materials: By selecting appropriate conditions, various new materials can be prepared. Simple equipment and low cost: The sol-gel method is suitable for industrial production, with simple equipment and low cost.

The disadvantages of the sol-gel method include the following:

Expensive raw materials: The raw materials used are relatively expensive, some of which are organic and harmful to health.

Long process time: Usually, the whole sol-gel process takes a long time, usually several days or weeks.

Volume shrinkage and cracking: During the drying process, there are a lot of micropores in the gel, which will release many gases and organic substances, leading to shrinkage and cracking.

Health risks: Some raw materials are organic and harmful to health [54,55].

### 2.3. Hydrothermal Synthesis of Transition Metal- and Lanthanide-Doped Luminescent Materials

The hydrothermal method is a commonly used method for preparing luminescent materials. By conducting chemical reactions in a high-temperature and high-pressure hydrothermal environment, luminescent materials with specific structures and properties can be synthesized. The basic principle of the hydrothermal method is to dissolve and recrystallize insoluble or soluble substances in a sealed pressure vessel through a high-temperature and high-pressure hydrothermal environment, thereby obtaining the desired material [56].

In order to enhance the photocatalytic hydrogen evolution activity of ZnIn_2_S_4_, Shen S doped different transition metals (Cr, Mn, Fe, and Co) into the lattice of ZnIn_2_S_4_ to narrow the band gap. The photocatalytic evaluation showed that the photocatalytic activity of ZnIn_2_S_4_ doped with Mn was 20% higher than that of undoped ZnIn_2_S_3_, while the activity of ZnIn_2_S_4_ doped with Cr, Fe, and Co was poorer in the order of Cr > Fe > Co. Based on the comprehensive characterization results, the band structure of ZnIn_2_S_4_ doped with transition metals was depicted, indicating the different effects of transition metal doping on the photocatalytic activity of hydrogen evolution [57].

Sadetskaya obtained bare hydroxyapatite nanoparticles (Ca_10_(PO_4_)_6_(OH)_2_, HAp) and hydroxyapatite nanoparticles doped with cobalt, nickel, or copper through hydrothermal synthesis and fully characterized them by X-ray diffraction, Raman spectroscopy, Fourier transform infrared spectroscopy, and transmission electron microscopy techniques. The results indicated that doping can affect the bandgap. Using density functional theory (DFT) calculations, the electrical properties of hydroxyapatite were studied using functionals in local density approximation (LDA) and generalized gradient approximation (GGA). Two methods for quantum chemistry calculations in DFT were considered. They calculated the density of states of the prepared samples and studied the correlation between the calculated bandgap values and experimental values. The revealed patterns provided a new perspective for the selection of dopants for biocompatible pigments and scaffold materials with semiconductor properties [58].

Kaynar synthesized a series of strontium stannate (SrSnO_3_) doped with Dy^3+^ of different weight percentages (1, 2, 3, and 5) through hydrothermal reaction and analyzed them using X-ray diffraction (XRD), energy dispersive spectroscopy (EDS), environmental electron scanning microscopy (ESEM), photoluminescence (PL), and cathodoluminescence (CL), revealing that SrSnO_3_ undergoes phase transition at 270 K [59].

**Figure 5:**
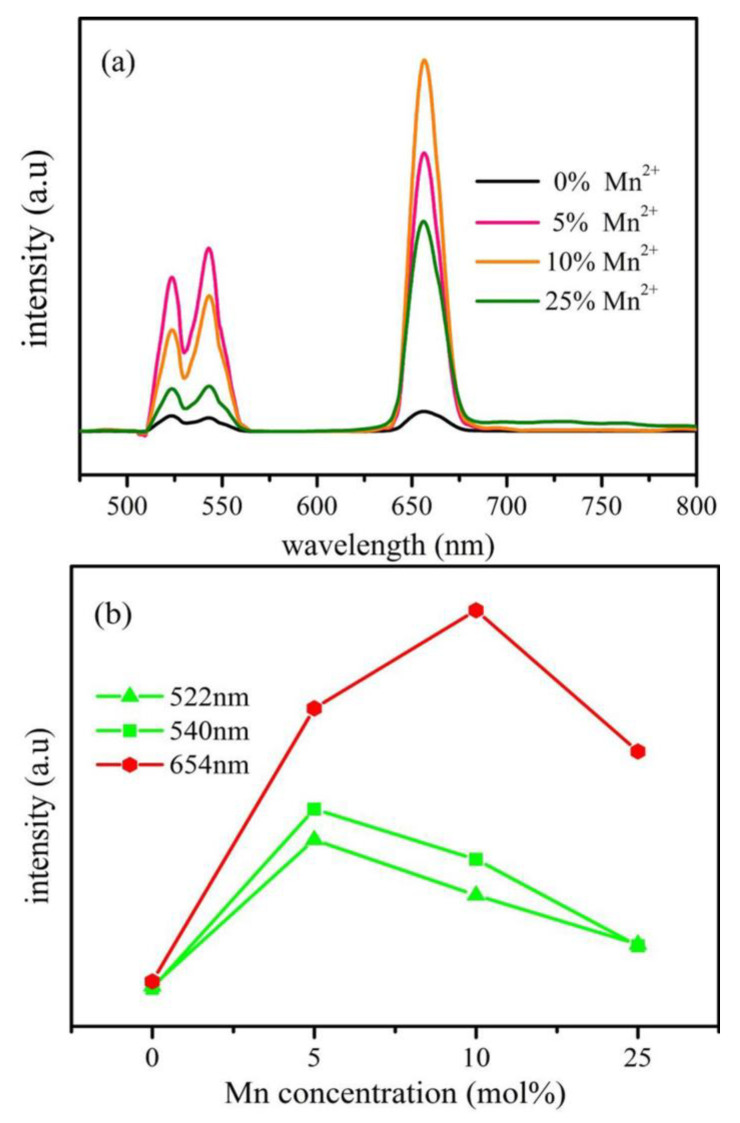
(**a**) Upconversion emission spectra of NaYF4:Yb^3+^, Er^3+^, and Mn^2+^ (0–25 mol%) nanoparticles under 980 nm excitation at room temperature and (**b**) intensity of green and red emissions as a function of Mn^2+^ ion concentration [60]. Copyright 2015. Reproduced with permission from Elsevier.

Hu Y reported an effective strategy for enhancing the red upconversion emission of NaYF_4_:Yb^3+^ and Er^3+^ nanocrystals by doping different concentrations of transition metal ions (Mn^2+^ or Fe^3+^). The upconversion emission spectrum of the sample showed a significant enhancement in red emission, and different transition metal ions had a significant impact on the red upconversion luminescence (Figure 5) [60].

Huang W prepared high-quality monodispersed LiYbF_4_ particles with octahedral shapes using a simple hydrothermal method and studied, in detail, the effects of the reaction temperature, reaction time, and molar ratio of EDTA to Yb^3+^ on the crystal phase and morphology of the prepared products. The upconversion (UC) luminescence properties of particles with an octahedral microstructure were studied under 976 nm excitation. The results indicated that the luminescence color of the corresponding product can be adjusted to bright green by changing the doping concentration of Er^3+^ [61].

Chavan synthesized SrMoO_4_ nanophosphate doped with dysprosium (Dy^3+^) using the hydrothermal method. The results indicated that the prepared SrMoO_4_ fluorescent powder has a tetragonal symmetric single-phase scheelite structure with grain sizes ranging from 10 to 50 nm. The photoluminescence spectrum showed blue emission at 485 nm and bright yellow emission at 576 nm under 353 nm excitation. The optimal concentration of Dy^3+^-doped SrMoO_4_ was found to be 6 mol% [62].

Reszczy ń ska obtained a series of Y^3+^, Pr^3+^, Er^3+^, and Eu^3+^-modified TiO_2_ photocatalysts through the sol-gel (SG) and hydrothermal (HT) methods. Spectral analysis showed that Pr^3+^-modified TiO_2_ can be excited under visible light in the range of 420–250 nm. In addition, the photocatalyst obtained by the HT method had higher photocatalytic activity and lower luminescence intensity than the photocatalyst prepared by the SG method. Luminescence properties of the samples, as well as XRD and XPS analyses, indicated that RE are in the form of their oxides, rather than in the form of cations in the crystal structure of TiO_2_ [63].

Zhou M synthesized M@CdSe/rGO composite materials through a simple one-step hydrothermal method, during which M-doped CdSe crystallization and GO reduction occurred simultaneously. The reduction of CdSe crystals doped with alkaline metal ions such as Mg/Ca/Sr/Ba and graphene oxide (GO) was achieved synchronously through the hydrothermal method. This method improved the aggregation and photo-corrosion of CdSe by introducing alkaline metal ions, such as Mg^2+^, Ca^2+^, etc., while utilizing the reduction of GO to enhance the conductivity and stability of the composite material. The obtained photocatalyst exhibited synergistically enhanced visible light catalytic activity and stability for the photodegradation of tetracycline hydrochloride (TC-HCl) solution, particularly by introducing 6 mol% Ca^2+^ and 5 wt% GO onto quantum dot CdSe. Under the action of 0.5 g/L catalyst, 15 mg/L TC-HCl was degraded by 85.6% within 1 h [64].

**Figure 6:**
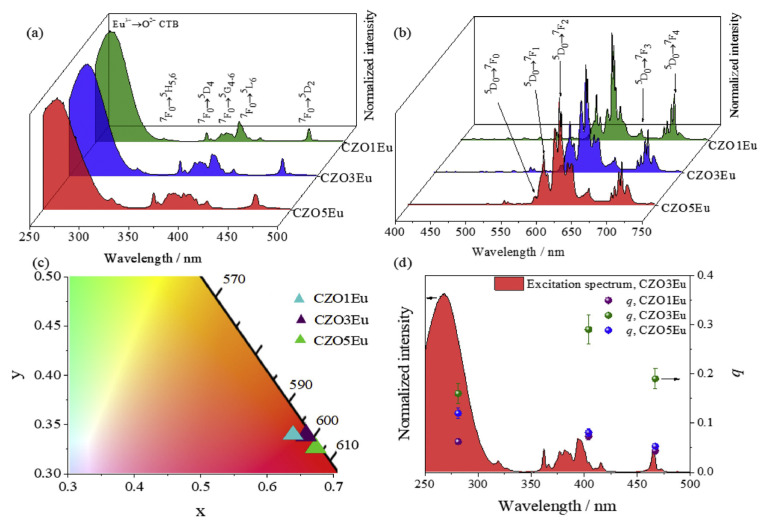
(**a**) Excitation spectra (298 K) excited at 613 nm, (**b**) emission spectra (298 K) excited at 260 nm, (**c**) 1931 Commission Internationale d’Eclairage (CIE) chromaticity diagram [29], and (**d**) absolute emission quantum yield (q) of the phosphors [65]. Copyright 2020. Reproduced with permission from Elsevier.

Macedo used the microwave-assisted hydrothermal method for the first time to synthesize europium doped with 1, 3%, and 5% CaZrO_3_ red luminescent phosphors. The excitation spectrum of the fluorescent powder showed the Eu^3+^f-f excitation bands (395 nm and 464 nm) and the Eu^3+^→O^2−^ charge transfer band at 250 nm, confirming that the CaZrO_3_ matrix acts as a sensitizer for Eu^3+^ luminescence. Similarly, the emission spectrum exhibited typical Eu^3+^ emission characteristics in the red spectral region, with CIE color coordinates of (0.642; 0.332), (0.663; 0.330), and (0.668; 0.327) for samples doped with 1%, 3%, and 5%, respectively, indicating high emission color purity (Figure 6) [65].

Zhao W successfully synthesized undoped ZnS and doped Zn_0.95−x_Co_0.05_Ni_x_S nanorods with different concentration ratios (x = 0.01, 0.03, and 0.05) by the hydrothermal method. UV–visible spectroscopy revealed the blue shift of the direct bandgap of co-doped ZnS nanorods. The PL spectrum showed a clear ultraviolet emission peak around 375 nm, and with the increase in Ni^2+^ doping concentration, there was a significant quenching phenomenon in the blue emission. All the samples synthesized by this method exhibited a single-phase wurtzite structure with good crystallization, as demonstrated by XRD studies, which indicated that all Co^2+^ and Ni^2+^ were successfully substituted for the lattice site of Zn^2+^ and generated single-phase Zn_0.95−x_Co_0.05_Ni_x_S [66].

A new type of transition metal-sensitized lanthanide near-infrared luminescent nanoparticles (CLNPs) was reported, using monoclinic Na_3_-CrF_6_ as both the matrix and sensitizer. Through efficient energy transfer from Cr^3+^ to lanthanide activators (Er^3+^, Tm^3+^, Yb^3+^, or Nd^3+^), its brightness was increased by up to 370 times compared to traditional lanthanide-sensitized nanoparticles [67].

The electrical neutrality compensation mechanism of multivalent dopants is essentially achieved through defect (vacancy), counterion, valence state adjustment, or carrier cancellation of charge imbalance. The core is to maintain the overall electrical neutrality of the lattice to stabilize the crystal structure. The electric neutral compensation mechanism directly affects the luminescence performance by stabilizing the valence state of luminescent ions, regulating the concentration of lattice defects, and optimizing the carrier balance and coordination environment. Reasonable compensation (such as appropriate vacancies and matched co-doping) can enhance luminescence intensity and stabilize luminescence color. However, excessive compensation or improper mechanisms (such as excessive defects and valence state fluctuations) can lead to luminescence quenching or a spectral shift. Therefore, precise control of the compensation mechanism is required when designing luminescent materials to achieve the desired luminescent performance.

The hydrothermal method can be carried out at relatively low temperatures, reducing the introduction of impurities. A high-temperature and high-pressure environment is conducive to the growth of crystals, resulting in high-quality crystals. By adjusting the reaction conditions, the morphology and size of the product can be controlled. However, special pressure vessels and heating equipment are needed. Equipment such as high-pressure reactors has high costs and complex operations. Precise control of temperature, pressure, and time is required, making the operation difficult [68].

### 2.4. Brief Summary

1. Low-temperature nanoparticle synthesis

The controllable synthesis of mixed-valence sulfides (such as Eu_3_S_4_ and EuSm_2_S_4_) by reacting dimethyl lanthanide metal halides with bis (trimethylsilyl) sulfides in an oil amine solvent stabilizes metastable-phase materials that are difficult to obtain at traditional high temperatures. Its magnetic semiconductor properties can be used for spin quantum devices.

2. Development of Multi-sulfur Oxide Matrix

Using BaZnOS as the substrate, lanthanide/transition metal dual doping can be achieved within 3 h at 950 °C, with a crystal structure that simultaneously accommodates two types of ions and regulates energy transfer.

3. Optimization of fluoride stability

By deprotonating ligands with cesium ions, the instability of fluoride acidic environments, such as NaLaF_4_^3+^, is solved, resulting in uniform nanorods with high near-infrared luminescence intensity [69].

The comparison of luminescent materials is shown in Table 1 and Table 2.

## 3. Application of Lanthanide/Transition Metal Ion-Doped Luminescent Materials

Rare earth ion- and transition metal ion-doped inorganic luminescent materials have attracted widespread attention due to their excellent optical properties. Compared to traditional organic dyes and quantum dots, rare earth/transition metal-doped inorganic luminescent materials have tunable luminescence, the ability to achieve near-infrared excitation and emission, low toxicity, excellent photostability, no photobleaching, and long luminescence lifetime. In addition, near-infrared emission has high tissue penetration depth and low autofluorescence and tissue scattering. Therefore, in recent years, breakthroughs have been made in the application research of rare earth/transition metal-doped inorganic luminescent materials, such as anti-counterfeiting, temperature sensing, optical information storage, lighting display, and biomedicine [70].

### 3.1. Application of Luminescent Materials in Solar Cells

The application of rare earth luminescent materials in solar cells is mainly reflected in improving the photoelectric conversion efficiency and expanding the spectral response range. The working principle of rare earth luminescent materials is based on two-photon or multiphoton processes. The luminescent center absorbs two or more photons, reaches the luminescent energy level through non-radiative relaxation, and then transitions to the ground state to emit visible photons. This mechanism enables rare earth luminescent materials to absorb low-energy photons and release high-energy photons, thereby improving the photoelectric conversion efficiency of solar cells [71,72].

#### 3.1.1. Crystal Silicon Solar Cell

Rare earth upconversion luminescent materials can absorb low-energy photons with energy smaller than the bandgap of crystalline silicon and release high-energy photons, thereby expanding the spectral response range of crystalline silicon solar cells and improving the photoelectric conversion efficiency [73,74,75].

Wang and his colleagues demonstrated the use of broadband Ce^3+^-sensitized quantum dots to improve the power conversion efficiency of mixed-crystal silicon solar cells [76]. Under UV excitation, the nanoparticles exhibit tunable near-infrared emission, and the measured quantum yield reaches 87%. Sun integrated quantum-cut nanoparticles into crystalline silicon solar cells, increasing the short-circuit current by 1.2 times and power conversion efficiency by 1.4 times. Recently, near-infrared luminescent materials doped with rare earth Yb^3+^ and Yb^3+^-Ln^3+^ ions have been selected as key technologies to improve the performance of silicon-based solar cells (SSCs).

#### 3.1.2. Perovskite Solar Cells

Rare earth-doped perovskite quantum dot materials can improve the photoelectric conversion efficiency of perovskite solar cells through energy transfer mechanisms. Jin et al. introduced YVO_4_:Eu/Bi nano-phosphors into the mesoporous TiO_2_/CH_3_NH_3_PbI_3_ perovskite solar cells (PSCs). Compared to the control device, these down-converted nano-phosphors improved the power conversion efficiency, which increased from 16.3% to 17.9%. In addition, devices based on m-TiO_2_/YVO_4_:Eu^3+^ and Bi^3+^ exhibited excellent stability under ultraviolet irradiation, maintaining an initial efficiency of 70% after 60 h of ultraviolet irradiation [77].

Song et al. reported that halide lead (CsPbCl_3_) PQDs (perovskite quantum dots) doped with Yb^3+^ ions exhibited highly effective quantum cutting emission at low excitation power [78]. This resulted in a significant increase of nearly 20% in PCE for SSCs. In addition, researchers achieved an extraordinary quantum yield of nearly 200% for near-infrared emission by changing the bandgap of the host crystal and introducing Pr^3+^ and Ce^3+^ ions with UV absorption properties [79]. Cai et al. demonstrated the use of Mn^2+^/Yb^3+^-doped CsPbCl_3_ perovskite nanocrystals as spectral converters to achieve down-conversion and down-frequency luminescence. When these perovskite nanocrystals are embedded in a polydimethylsiloxane matrix, they have been proven to be effective luminescent solar concentrators due to their strong light capture effect [80]. Considering the stability of perovskite itself, the research team led by Song designed PQDs@ZnS. The core-shell device effectively promotes the energy transfer of converting PeQDs to Yb^3+^ through ZnS coating. The presence of ZnS coating significantly improves the power conversion efficiency (PCE) of silicon solar cells, with a relative increase of 13.5% in PCE measured at 30 °C [81].

Rare earth luminescent materials have the characteristics of large absorption cross-section, high luminescence efficiency, long fluorescence lifetime, stable photophysical output, and strong spectral modulation. These characteristics enable rare earth luminescent materials to effectively absorb and convert light energy in solar cells, improving photoelectric conversion efficiency.

In summary, the application of rare earth luminescent materials in solar cells is mainly achieved by improving the photoelectric conversion efficiency and expanding the spectral response range. Their application in crystalline silicon and perovskite solar cells has demonstrated significant effects and potential.

### 3.2. Application of Luminescent Materials in Night Vision Devices

The application of luminescent materials in night vision devices is mainly reflected in infrared light-emitting diodes (IR LEDs). Infrared light-emitting diodes can emit infrared light, which is not visible in the visible light range but is very important in night vision devices. Infrared light can be received by special photoelectric sensors and converted into visible light images, enabling imaging in nighttime or low-light environments. Infrared light-emitting diodes are commonly used in devices such as night vision goggles and thermal imagers to assist users in observing and monitoring at night or in low-light conditions. According to current reports, most near-infrared phosphors with emission wavelengths in the range of 750 to 1000 nm have the potential for application in night vision imaging.

Tu reduced the crystal field intensity and shifted the emission spectrum towards lower energy by increasing the Cr^3+^ doping concentration. As the concentration of Cr^3+^ increased, the relative intensity of the emission peak gradually increased, and the emission spectrum gradually widened. At 373 K, the integrated emission intensity of the sample can be maintained at 86% of room temperature, demonstrating competitive luminescent thermal stability. AGO(Al_2_._26_Ge_0_._74_O_4_._87_):Cr^3+^ material and a blue LED chip were packaged into near-infrared pc LED devices, which showed great potential in night vision applications [82].

Researchers from China University of Geosciences (Beijing) and Peking University synthesized K_3_ScF_6_:Cr^3+^ phosphors with a cubic structure using the hydrothermal method. These phosphors can be effectively excited by blue light (430 nm) and red light (630 nm), producing broadband Cr^3+^ emission from 650 nm to 950 nm, with a maximum full width at half maximum of 430 nm. At 150 °C, they also exhibited good thermal stability and maintained 87.3% of their initial strength. At a driving current of 100 mA, their photoelectric efficiency can reach 9.315% of the manufactured NIR-LED. Therefore, NIR-LED devices based on this phosphor can be implemented in night vision imaging systems [83].

Das S prepared Cr^3+^-doped Zn_3_Ga_2_Ge_2_O_10_ long afterglow material using the solid-state reaction method. This material emitted near-infrared long afterglow and was used as a secret light source for night vision devices. The developed materials can be used for marking, tracking, and positioning purposes in national defense applications [84].

**Figure 7:**
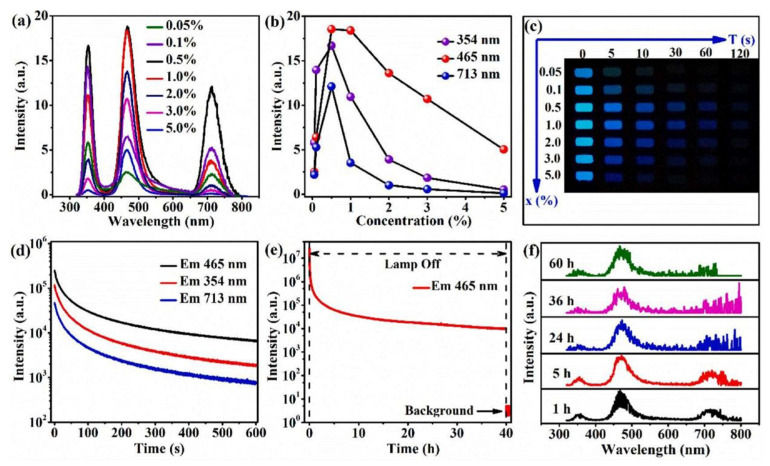
(**a**) PersL spectra of SYGO:x%Bi^3+^ (x = 0.05–5.0) samples. (**b**) Relationship between the integral PersL intensity and Bi^3+^ doping concentration. (**c**) PersL images of SYGO:Bi^3+^ phosphors taken after irradiation by a 254 nm UV lamp for 5 min. (**d**) PersL decay curves of SYGO:Bi^3+^ by monitoring the three PersL wavelengths. (**e**) PersL decay curve of SYGO:Bi^3+^ by monitoring the emission at 465 nm after illumination by a 254 nm UV lamp for 10 min (recorded for 40 h). (**f**) PersL spectra of the SYGO:0.5%Bi^3+^ sample at different delay times after ceasing the excitation source [85]. Copyright 2021. Reproduced with permission from Elsevier.

Zhou Z reported the development of a novel sunlight-activated PersL material based on a Bi^3+^-doped Sr_3_Y_2_Ge_3_O_12_ (SYGO) phosphor, which exhibited strong UV–visible NIR PersL and ultra-long PersL over 60 h. This newly developed PersL material, due to its high response to direct and diffuse sunlight radiation, can effectively be repeatedly activated by sunlight radiation under various weather conditions. By utilizing the unique PersL and PSL characteristics, researchers demonstrated the application of SYGO:Bi^3+^ in night vision identification (Figure 7) [85].

### 3.3. Biological Imaging and Biosensing

#### 3.3.1. Luminescent Transition Metal Complexes as Probes for Cell Viability

Assessing cell viability plays a crucial role in biomedical, pathological, and scientific investigations. While conventional approaches depend on colorimetric assays, fluorescent labeling techniques offer enhanced precision and detailed insights into cellular conditions. Such methods enable real-time observation of individual cell viability through fluorescence microscopy, while flow cytometric analysis permits precise quantification of living cells and discrimination between subpopulations with varying physiological states.

Orellana’s team synthesized a novel luminescent probe, (dibenzo[h,j]dipyrido[3,2-a:2′,3′-c]phenazine)bis(2,2′-bipyridine)ruthenium(II) dication (RB2Z), demonstrating selective discrimination between viable and nonviable cells [86]. The complex exhibits distinct membrane permeability characteristics, namely, being excluded by intact cellular membranes but readily accumulating in the nuclei of membrane-disrupted dead cells. This differential uptake mechanism establishes RB2Z as a reliable marker for necrotic cell identification within heterogeneous populations.

In parallel research, Vaidyanathan and Nair’s group designed a platinum(II)-based complex incorporating imidazolyl terpyridine and benzimidazolyl terpyridine ligands with similar membrane selectivity [87]. Their impermeable metalloprobe specifically targets nuclei in membrane-compromised cells, proving particularly valuable for monitoring lead protein-triggered apoptotic progression. Through fluorescence imaging, the complex effectively captured hallmark apoptotic morphological alterations, including plasma membrane blebbing and chromatin fragmentation.

Li and his colleagues designed an iridium (III) solvated complex (a cyclometalated iridium(III) solvent complex [Ir(pdz)_2_(H_2_O)_2_] + [OTf] − (IrC1)) as a luminescent indicator for cell death. This complex exhibits selective labeling of dead cell nuclei, as its cellular and nuclear uptake increases during membrane permeation, allowing flow cytometry to distinguish live cells, early apoptotic cells, and dead cells based on their different membrane and nuclear membrane permeabilities [88].

Chao’s research team designed a novel platinum(II)-based complex with dual emission properties (specifically, an organoplatinum(II) complex [Pt(C^N^N)(Cl)], where C^N^N = 5,6-diphenyl-2,2′-bipyridine, denoted as Pt1) for two-photon bioimaging applications. In aqueous solution, this complex displayed both fluorescence (emission peak at 445 nm) and phosphorescence (emission peak at 573 nm), arising from monomeric species and self-assembled supramolecular nanostructures, respectively. During cell death, compromised plasma membrane integrity enhanced cellular uptake and accumulation of the complex, triggering its intracellular self-assembly and increasing the 573 nm/445 nm emission ratio. This ratiometric response serves as an indicator of membrane damage severity, enabling discrimination among viable, apoptotic, and necrotic cells. Additionally, the complex was successfully applied to track inflammatory processes in zebrafish models [89].

Li, Yam, and co-workers developed a cationic platinum(II) complex (with counterions including CF_3_SO_3_^−^, PF_6_^−^, ClO_4_^−^, and BF_4_^−^) capable of assessing cell viability through distinct subcellular distribution patterns in living versus dead cells. At higher concentrations, the complex underwent self-assembly via Pt···Pt and π-π stacking interactions, forming well-defined nanostructures accompanied by an emission shift from green (λ_em_ = 490 and 520 nm) to red (λ_em_ = 550–800 nm). In viable cells, the complex is primarily localized in lysosomes as monomeric species, emitting green fluorescence. However, upon cell death—marked by nuclear membrane permeabilization—the complex readily translocated into the nucleus, where it aggregated into red-emitting nanostructures. This dynamic shift in subcellular localization and accompanying emission transition enabled real-time monitoring of acid-induced necrosis in HeLa cells [90].

#### 3.3.2. Luminescent Transition Metal Complexes as Probes for Bacterial Cells

Bacterial infections remain a significant threat to global health, ranking as the second leading cause of mortality worldwide. While antibiotics have traditionally been effective against such infections, their overuse and misuse have accelerated the rise of antibiotic-resistant bacterial strains. This growing resistance poses serious challenges in treating infections, particularly those caused by multidrug-resistant pathogens. Consequently, the selective identification of pathogenic bacteria holds crucial clinical value, enabling precise diagnosis and targeted therapeutic interventions for bacterial infections [91].

Ding, Yu, and their research team developed an innovative approach for bacterial detection using metal complexes. They designed a cationic platinum(II) complex that serves as a sensitive LPS probe through its unique photophysical response. Upon LPS binding, the complex undergoes distinct molecular changes, namely, strengthened Pt···Pt metallophilic interactions and enhanced π-π stacking, which activate a previously restricted 3MMLCT transition, resulting in intense deep-red emission at 650 nm. This specific LPS recognition mechanism allowed for rapid wash-free discrimination of Gram-negative (*E. coli*) from Gram-positive bacteria (*S. aureus* and *B. subtilis*) within minutes. Expanding their detection platform, the researchers also developed a series of aggregation-induced emission (AIE)-active iridium(III) complexes ([Ir(C^N)_2_(N^N)]Cl_2_, Ir1–Ir3) featuring quaternary ammonium groups. These iridium complexes demonstrated excellent performance in no-wash bacterial imaging and LPS detection, offering a complementary approach to their platinum-based system. The dual-complex strategy provides versatile tools for rapid pathogen identification in clinical and research settings [92].

Xu, Wang, and their research team developed a series of three positively charged AIE-active cyclometalated iridium(III) photosensitizers (Ir1-3) for targeted Gram-positive bacterial detection and eradication. These innovative complexes exhibit unique aggregation-induced emission properties upon interaction with lipoteichoic acid (LTA), forming highly luminescent aggregates that produce intense fluorescence signals, specifically in Gram-positive bacterial strains, including Bacillus subtilis, *Staphylococcus aureus*, and methicillin-resistant *S. aureus* (MRSA). The complexes demonstrate remarkable specificity, enabling clear discrimination among different microbial species through their selective emission activation patterns. This allows for reliable differentiation between Gram-positive *S. aureus*, Gram-negative *E. coli*, and fungal *C. albicans*. Beyond their diagnostic capabilities, these iridium(III) complexes also serve as effective photosensitizers, combining selective bacterial imaging with potent antimicrobial activity for a comprehensive theranostic approach against Gram-positive pathogens [93].

Sassal et al. synthesized three cationic iridium(III) complexes with aggregation-induced emission (AIE) properties, specifically cyclometalated polypyridine structures of the form [Ir(PQ)_2_(N^N)]Cl(1-3), where PQ = 2-phenylquinoline and N^N = 2,2′-bipyridine derivatives. These compounds were designed for the dual-purpose detection and eradication of drug-resistant bacterial strains. Upon interaction with bacteria, the complexes engage with lipoteichoic acid (LTA) or lipopolysaccharide (LPS) via electrostatic and hydrophobic forces, leading to a blue shift in emission, along with increased fluorescence intensity. Additionally, the compounds demonstrated potent antibacterial effects against resistant pathogens, including carbapenem-resistant *Acinetobacter baumannii* and methicillin-resistant *Staphylococcus aureus* [94].

Wang, Zhou, and their research team synthesized a novel ruthenium(II) polypyridine complex (Ru2) featuring dual alkynyl groups, specifically engineered for selective Gram-positive bacterial imaging. This innovative compound targets amino phospholipids—including phosphatidylethylethanolamine and lysylphosphatidylglycerol—that are prominently expressed on Gram-positive bacterial membranes, achieving selective binding through amine–alkyne click chemistry. The Ru2 complex demonstrated precise discrimination capabilities, enabling both fluorescent labeling and photodynamic eradication of pathogenic Gram-positive strains like *Staphylococcus aureus* and its drug-resistant variants, while exhibiting excellent biocompatibility with mammalian cells [95].

#### 3.3.3. Luminescent Transition Metal Complexes as Probes for Microenvironment

The intracellular microenvironment is controlled by various factors, including O_2_ levels, pH values, polarity, viscosity, and temperature. These factors play a crucial role in controlling the physical and chemical behavior of cellular components, such as their diffusion, transport, and intermolecular interactions. The interference of these factors can lead to cellular dysfunction and is closely related to the development of serious diseases. Therefore, the development of luminescent probes that can provide a deep understanding of the pathophysiological microenvironment has enormous potential in assisting in the diagnosis and treatment of clinical diseases [96,97].

Recent advances in viscosity-sensitive probes have enabled real-time monitoring of liquid–liquid phase separation (LLPS) in living cells—a fundamental mechanism driving the formation of membraneless organelles and implicated in various physiological and pathological processes [98]. Zhang et al. engineered an innovative iridium(III)-based molecular probe (Ir–OMe) that exhibits remarkable viscosity-dependent photophysical properties. This probe demonstrated a dramatic increase in emission lifetime from 163.39 ns to 1023.90 ns when environmental viscosity rose from 1.45 to 165.47 cP. Such exceptional sensitivity enabled precise tracking of FUS protein phase separation dynamics in NIH/3T3 cells through phosphorescence lifetime imaging microscopy (PLIM). Notably, the probe’s emission lifetime showed progressive elongation during light-induced phase transitions, quantitatively reflecting the growing size and viscosity of protein condensates [99]. In a parallel work, Tan, Zhang, Li, Mao, and collaborators designed a structurally sophisticated ruthenium(II) complex (Ru1) featuring dual TPP+ moieties for simultaneous induction and monitoring of DNA phase separation. The complex’s cationic triphenylphosphonium groups significantly enhanced DNA binding affinity, triggering DNA condensation. This structural transition was accompanied by measurable increases in emission lifetime, permitting real-time visualization of nuclear DNA phase separation in A549 cells via two-photon PLIM. Beyond its diagnostic utility, this multifunctional agent demonstrated potent anticancer effects by modulating gene expression patterns and chromatin organization, showing efficacy in both cellular and animal models [100]. In a parallel work, Tan, Zhang, Li, Mao, and collaborators designed a structurally sophisticated ruthenium(II) complex (Ru1:[Ru(phen-PPh_3_)_2_(dppz)](NO_3_)_4_; phen-PPh_3_ = (6-(1,10-phenanthroline-5-carboxamido)hexyl)-triphenylphosphonium; phen = 1,10-phenanthroline))) featuring dual TPP+ moieties for simultaneous induction and monitoring of DNA phase separation. The complex’s cationic triphenylphosphonium groups significantly enhanced DNA binding affinity, triggering DNA condensation. This structural transition was accompanied by measurable increases in emission lifetime, permitting real-time visualization of nuclear DNA phase separation in A549 cells via two-photon PLIM. Beyond its diagnostic utility, this multifunctional agent demonstrated potent anticancer effects by modulating gene expression patterns and chromatin organization, showing efficacy in both cellular and animal models [100].

### 3.4. Non-Contact Temperature Measurement

Accurately measuring the temperature of microelectronic devices, biological cells, and high-speed moving objects demands sensors with rapid response times and fine spatial resolution, which conventional sensing technologies fail to achieve [101,102,103,104,105]. Consequently, there is a critical need to advance non-contact temperature sensing solutions capable of high thermal sensitivity and swift detection. Compared to conventional contact-based thermometry, optical temperature sensing offers significant benefits, including instantaneous response, precise spatial mapping, and remote measurement capabilities [106,107,108,109,110,111]. Researchers have currently validated multiple luminescent thermometry mechanisms to address these requirements.

#### 3.4.1. Reading Based on the Luminous Intensity Ratio

**Figure 8:**
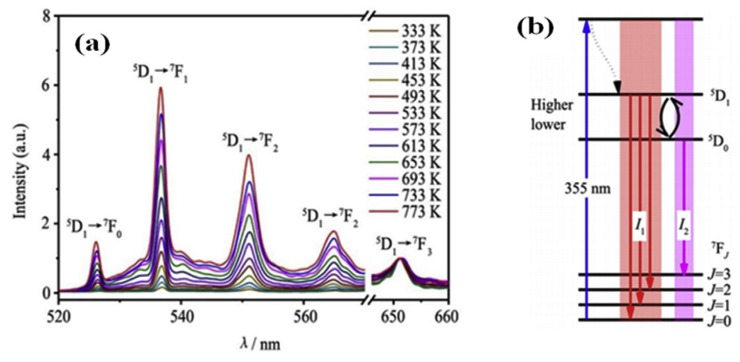
(**a**) Temperature-dependent emission spectra of 5D1 and 5D0 in YBO_3_:2% Eu3t. (**b**) Temperature sensing mechanism [115]. Copyright 2021. Reproduced with permission from Elsevier.

Trivalent europium ions (Eu^3+^), with their 4f^6^ electron configuration, serve as one of the most efficient red-emitting activators in light-emitting diodes (LEDs) [112,113,114]. Temperature monitoring using Eu^3+^-doped phosphors is particularly important, as the thermal behavior of LEDs can be analyzed by examining the temperature-dependent photoluminescence (PL) properties of these phosphors [115]. For instance, L. Zhao et al. explored YBO_3_:2% Eu^3+^ as a temperature probe by utilizing the fluorescence intensity ratio (FIR) technique [116,117]. The yttrium borate (YBO_3_) phosphor, doped with europium, was prepared via solid-state reaction. The crystalline structure and surface morphology of the synthesized material were examined using X-ray diffraction (XRD) and scanning electron microscopy (SEM). Under 355 nm excitation, the 2% Eu^3+^-doped sample exhibited distinct luminescence behavior, as illustrated in Figure 8a,b. Notably, the intensity ratio between the thermally coupled levels (^5^D_1_ and ^5^D_0_) showed a progressive increase with rising temperature.

Hua et al. investigated the thermometric performance of Er^3+^-doped LaBaMoO_6_ within the 303–483 K range by employing the luminescence intensity ratio (LIR) method. Their results demonstrated a maximum relative sensitivity (S_a_) of 0.017 K^−1^ at 483 K [118].

M. Runowski et al. developed a highly sensitive and precise thermometric approach capable of detecting extreme temperatures up to 1000 K, utilizing Yb^3+^-Tm^3+^ co-doped YVO_4_ nanostructures. This system addresses the challenges typically encountered in high-temperature monitoring. Temperature detection is achieved by analyzing both thermally coupled levels (TCLs) and non-TCLs of Tm^3+^ and Yb^3+^ ions in the red and near-infrared spectral regions. By employing the emission intensity ratio of Tm^3+^ TCLs (700/800 nm), the researchers achieved a record-high sensitivity of 2.9% K^−1^ at 300 K [119].

#### 3.4.2. Temperature Sensing Through Luminescence Lifetime

Cao et al. reported the thermometric properties of Sm^2+^-doped SrB_4_O_7_, demonstrating its potential for temperature sensing applications [120]. Separately, Li et al. investigated the thermal response of Eu^3+^-activated BaY_2_ZnO_5_, utilizing the rise time of its luminescence for temperature detection across the 330–510 K range [121].

#### 3.4.3. Optical Temperature Measurement Based on Temperature-Induced Spectroscopy

**Figure 9:**
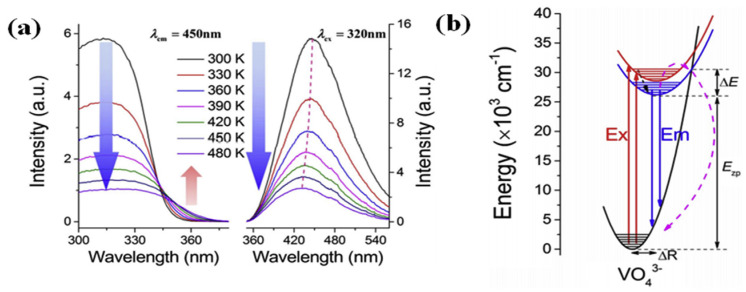
(**a**) Temperature-dependent excitation (left) and emission spectra (right). (**b**) Configurational coordinate diagram for the emission and excitation of the VO_4_^3−^ [122]. Copyright 2017. Reproduced with permission from OSA.

Zhou et al. explored ratiometric temperature sensing by monitoring the thermally induced redshift of the charge transfer band (CTB) in GdVO_4_ phosphors [122]. As depicted in Figure 9a (left), the excitation spectra exhibit clear temperature dependence. Conversely, the emission spectra under 320 nm UV excitation (Figure 9a (right)) display an opposite thermal response, with a blueshift (shorter wavelengths) at elevated temperatures (indicated by dotted lines). Notably, the excitation intensity near 360 nm plays a key role in the CTB redshift, showing a monotonic increase with temperature. These thermally induced shifts in both photoluminescence excitation (PLE) and emission (PL) can be explained using the configurational coordinate diagram presented in Figure 9b.

### 3.5. Anti-Counterfeiting Applications

The emission color of luminescent materials is crucial for encoding information, which is used for anti-counterfeiting and optical multiplexing [123]. UCNPs based on Ln^3+^ are highly suitable for this application due to their long luminescence lifetime, large anti-Stokes shift, and distinguishable spectral fingerprints [124,125]. However, despite these significant advances, traditional UCNPs have limited information storage capacity and complexity in preventing forgery [126,127]. TM^n+^ has become a competitive alternative to Ln^3+^ dopants in terms of time encoding due to its significantly longer lifetime [128]. For example, Liu et al. demonstrated that combining long-lived Mn^2+^ upconversion emission with relatively short-lived lanthanide upconversion emission enables the development of binary time codes for efficient data encoding in multi-level anti-counterfeiting. In their study, they demonstrated that UCNPs with a binary color order can be obtained by spatially controlling Mn^2+^ co-doping into the hexagonal NaLnF_4_ lattice. Transition metal ions with a 3d^5^ configuration exhibit long-lived excited states due to their stable semi-full electron configuration and spin-forbidden transitions and can adapt to the structure and charge environment of lanthanide matrix lattices. Therefore, they can serve as luminescent centers with long-lived emission characteristics in such matrices and have important applications in fields such as phosphors and phosphorescent materials (such as Mn^2+^-doped YAG phosphors). Due to the transition properties of transition metals with a 3d^5^ configuration that prohibit rotation, it can serve as a long-lived emission center for lanthanide matrix lattices. When Mn^2+^ dopants are excited at 980 nm, their dual-mode luminescence at 531 nm has a lifetime of over 30 milliseconds. The long lifetime of dual-mode Mn^2+^ luminescence contributes to the establishment of various up- and down-conversion time-domain optical properties for multi-layer anti-counterfeiting and multiple biological labels [129,130].

## 4. Conclusions and Outlook

### 4.1. Advantages of Rare Earth and Transition Metal-Doped Luminescent Materials

#### 4.1.1. Excellent Optical Performance

Rare earth and transition metal-doped luminescent materials have characteristics such as long fluorescence lifetime, narrow spectral lines, and tunable fluorescence emission wavelength. These characteristics make them potentially applicable in areas such as fluorescent biomarkers, giving them the potential to become a new generation of fluorescent biomarker materials to replace molecular probes. In addition, they also have the characteristics of tunable luminescence, near-infrared excitation and emission, low toxicity, excellent photostability, and no photobleaching [131,132].

#### 4.1.2. Near-Infrared Luminescence Characteristics

These materials have bright near-infrared luminescence, which makes them widely applicable in fields such as displays, lighting devices, and biological imaging technology. The near-infrared luminescence characteristics make them excellent in biological imaging technologies, such as fluorescent probes and biological tissue imaging [133].

#### 4.1.3. High Conversion Efficiency and Long Lifespan

Rare earth luminescent materials typically have high energy conversion efficiency, which can effectively convert absorbed light energy into visible light or other forms of energy. In addition, they have a long fluorescence lifetime and can maintain stable luminescence under continuous excitation, which makes them perform well in applications where they emit light for a long time [134].

#### 4.1.4. Multiple Application Scenarios

Rare earth and transition metal-doped luminescent materials have wide applications in various fields, including anti-counterfeiting, temperature sensing, optical information storage, lighting display, and biomedical applications. For example, temperature detection based on the luminescence intensity ratio of inorganic luminescent materials can effectively avoid the limitations of traditional contact thermometers and achieve non-contact temperature measurement. In addition, these materials can also be used to manufacture high-performance and long-life displays and lighting devices, as well as energy conversion and storage, such as electrode materials for solar cells and batteries [135].

### 4.2. Challenges Faced by Rare Earth and Transition Metal-Doped Luminescent Materials

#### 4.2.1. High Preparation Cost

The preparation cost of luminescent materials doped with rare earth and transition metals is very high. In addition, the preparation process of luminescent materials also requires special conditions, such as high temperature, which will increase the preparation cost [136].

#### 4.2.2. Shortage of Resources

Rare earth elements are important raw materials for preparing rare earth upconversion luminescent materials, but rare earth resources are extremely limited. Currently, over 90% of the world’s rare earth reserves are concentrated in China, and other countries have almost no rare earth resources. Due to the shortage of rare earth resources and the continuous rise in prices, their widespread application is restricted [137].

#### 4.2.3. Toxicity of Elements

Although rare earth upconversion luminescent materials can achieve high-efficiency luminescence, the rare earth elements contained in them are toxic. Long-term exposure can cause harm to the human body; therefore, safety precautions should be taken during use. Meanwhile, the production and processing of rare earth elements can also cause environmental pollution problems [138].

#### 4.2.4. Color Saturation Is Not as Good as Direct Luminescence

Although luminescent materials can achieve colorful effects, their color saturation is not as good as materials that directly emit light. Therefore, in application scenarios that require high color saturation, the application of rare earth and transition metal-doped luminescent materials still has limitations [139].

In summary, although rare earth and transition metal-doped luminescent materials have a wide range of applications in LED, photovoltaic, and display fields, they also have some drawbacks, such as high preparation costs, resource shortages, toxicity of elements, and lower color saturation compared to direct luminescence. During use, it is necessary to carefully consider these issues and further improve material research and preparation technology to promote its development in practical applications.

### 4.3. Future Directions

Although rare earth and transition metal-doped luminescent materials have many advantages, they still face some challenges. Future research will focus on the following areas: By improving synthesis technology, the purity and crystallinity of materials can be enhanced to ensure repeatability and high quality. Better near-infrared luminescence performance can be achieved by exploring new combinations of rare earth transition metal ions. The potential of application areas, especially in the fields of new energy, environmental protection, and biomedical sciences, may be further expanded and explored [140]. Overall, rare earth transition metal ion-doped near-infrared luminescent materials are advanced materials with broad application potential. By deeply understanding their characteristics, optimizing their preparation methods, and expanding their application fields, we can further explore their potential and bring more convenience and possibilities to our lives. Future research will focus on exploring new combinations of rare earth transition metal ions to achieve better near-infrared luminescence performance, as well as their applications in new energy, environmental protection, and biomedical fields.

## Figures and Tables

**Table 1:** Comparison between lanthanide ion-doped oxides and transition metal ion-doped oxides.

Performance Criteria	Lanthanide Ion-Doped Oxides	Transition Metal Ion-Doped Oxides
Luminescence mechanism	Mainly f-f transitions within the 4f electron layer (some containing f-d transitions, such as Ce^3+^ and Eu^2+^), which are shielded by outer electrons and less affected by the matrix	Mainly the d-d transition or charge transfer transition of 3d electrons (such as between Mn^4+^ and ligands); 3d electrons have no outer shielding and are significantly affected by crystal fields
Spectral characteristic	Narrowband emission (half width usually <50 nm), fixed wavelength (such as Eu^3+^ red light~615 nm and Tb^3+^ green light~545 nm), and high color purity	Broadband emission (peak width at half maximum mostly >100 nm), with wavelength varying with the intensity of the matrix crystal field (e.g., Cr^3+^ can emit red to near-infrared light)
Quantum efficiency	When there is no spin barrier, the f-d transition quantum efficiency of Ce^3+^ approaches 100%. The f-f transition has a slightly lower spin barrier, but it is still higher than most TM ions	Moderate (d-d transitions are mostly spin-forbidden), such as Cr^3+^ with a quantum efficiency of about 70% in Al_2_O_3_, which is greatly affected by defect quenching

**Table 2:** Comparison of synthesis methods.

Synthesis Method	Key Technical Points	Performance Advantages
High-temperature solid-phase synthesis method	Raw material pretreatment, homogenization of mixed materials, and precise control of high-temperature reactions	Suitable for refractory materials (such as magnesia alumina spinel); it can maintain structural stability at high temperatures above 1600 °C and has strong chemical inertness
Sol-gel method	Low-temperature hydrolysis (<100 °C) forms a uniform sol, combined with heat treatment doping	Nanoparticle size deviation < 5%; controllable composition
Hydrothermal/solvothermal method	Crystallization at 120–200 °C regulates crystallinity and enhances photocatalytic activity	Improves material crystallinity and stability (photocatalytic efficiency increased by 3.7 times)
